# An Overview of Recent Advances in the Neuroprotective Potentials of Fisetin against Diverse Insults in Neurological Diseases and the Underlying Signaling Pathways

**DOI:** 10.3390/biomedicines11112878

**Published:** 2023-10-24

**Authors:** Xiangwen Tang, Peng Deng, Yizhen Jiang, Lingling Zhang, Yuqing He, Hao Yang

**Affiliations:** 1 Translational Medicine Center, Hong Hui Hospital, Xi’an Jiaotong University, Xi’an 710054, China; tangxiangwen1020@163.com (X.T.); sxjxzhangll@126.com (L.Z.); 2 Basic Medical School Academy, Shaanxi University of Traditional Chinese Medicine, Xianyang 712046, China; dengpeng0908@126.com (P.D.); j17709158976@126.com (Y.J.); 3 School of Basic Medical Sciences, Ningxia Medical University, Yinchuan 750004, China; yuqing572817@163.com

**Keywords:** fisetin, neurological diseases, flavonoid, neuroprotection, nerve injury

## Abstract

The nervous system plays a leading role in the regulation of physiological functions and activities in the body. However, a variety of diseases related to the nervous system have a serious impact on human health. It is increasingly clear that neurological diseases are multifactorial pathological processes involving multiple cellular systems, and the onset of these diseases usually involves a diverse array of molecular mechanisms. Unfortunately, no effective therapy exists to slow down the progression or prevent the development of diseases only through the regulation of a single factor. To this end, it is pivotal to seek an ideal therapeutic approach for challenging the complicated pathological process to achieve effective treatment. In recent years, fisetin, a kind of flavonoid widely existing in fruits, vegetables and other plants, has shown numerous interesting biological activities with clinical potentials including anti-inflammatory, antioxidant and neurotrophic effects. In addition, fisetin has been reported to have diverse pharmacological properties and neuroprotective potentials against various neurological diseases. The neuroprotective effects were ascribed to its unique biological properties and multiple clinical pharmacological activities associated with the treatment of different neurological disorders. In this review, we summarize recent research progress regarding the neuroprotective potential of fisetin and the underlying signaling pathways of the treatment of several neurological diseases.

## 1. Introduction

With the acceleration of population aging and pace of life, the prevalence of neurological diseases is sharply increasing, and neurological diseases have become one of the leading causes of disability and mortality worldwide [1], which poses a huge threat to human health. The etiology of neurological diseases is intertwined with multiple genetic, aging and environmental factors, and thus, its pathogenesis is very complex and generally involves a variety of molecular mechanisms. Although most neurodegenerative diseases such as Alzheimer’s disease (AD), Parkinson’s disease (PD), Huntington’s disease (HD), amyotrophic lateral sclerosis (ALS), epilepsy and dementia, traumatic brain injury (TBI), stroke (ischemic or hemorrhagic) and other diseases have specific initiation mechanisms and pathologies other than the core mechanism, certain identical core mechanisms are seemly contributable to the initiation of most neurological diseases [2]. Until now, numerous treatment strategies for a single pathway and specific mechanisms of neurological diseases are not ideal. Therefore, it is necessary to find an effective approach for targeting multiple pathological processes and a variety of mechanisms.

In recent years, flavonoids derived from the natural diet have attracted the utmost attention of scientists for their extensive therapeutic potentials, including anti-inflammatory, antioxidant, anticarcinogenic, antiviral, antibacterial, neurotrophic, neuroprotective and immune responses [1,3,4]. The flavonol fisetin is conventionally described as 2-(3,4-dihydroxyphenyl)-3,7-dihydroxy-4H-1-benzopyran-4-one, 3,3′,4′,7-tetrahydroxyflavone, or 5-deoxyquercetin, and has been identified as a secondary metabolite in numerous green plants, particularly fruits, barks and hardwoods [5]. Importantly, these plants are easily accessible. To date, numerous studies have shown that flavonoids yield broad therapeutic effects in mitigating several neurological diseases, including the above-mentioned neurodegenerative diseases and other central nervous system (CNS) injuries. In the comprehensive pool of flavonoids, fisetin, a class of bioactive natural hydrophobic flavonols, is found abundantly in various fruits and vegetables such as strawberry, apple, mango, persimmon, kiwi fruit, grape, tomato, onion, kale and cucumber [5,6]. Due to the multiple physiological benefits of fisetin, including antioxidant, antiviral, anti-inflammatory, anticarcinogenic, antibacterial, neurotrophic, neuroprotective, and immune-stimulant behaviors, there is a growing interest in the pharmacological effects of these plant-derived fisetin against various neurological diseases. In the current review, we will focus mainly on a detailed and dedicated outline of the therapeutic potential of fisetin in treating neurological health complications and a variety of acute and chronic neurodegenerative diseases, including AD, PD, HD, epilepsy, ALS, TBI, stroke, schizophrenia and other diseases. In addition, the detailed cellular mechanisms and exhaustive molecular pathways in treating these complex, multifaceted disease processes are systemically discussed to better understand the therapeutic effects of fisetin (Table 1). In addition, many more aspects of neuroprotection of fisetin against the abovementioned neurological diseases are further discussed here than in previous reports. The review also provides new combined therapeutic strategies for the comprehensive treatment of neurological injuries and neurodegenerative disorders with plant-derived flavonoids. More importantly, this review critically provides the molecular mechanisms of fisetin therapy for a variety of neurological disorders and significant evidence for the development of new drugs. Hopefully, this review will lay the foundation for further research on the treatment of neurological diseases with fisetin, but also provide evidence for the development of new drugs.

## 2. Fisetin Structure, Physicochemical Attributes, Pharmacokinetics and Toxicity

**Figure 1:**
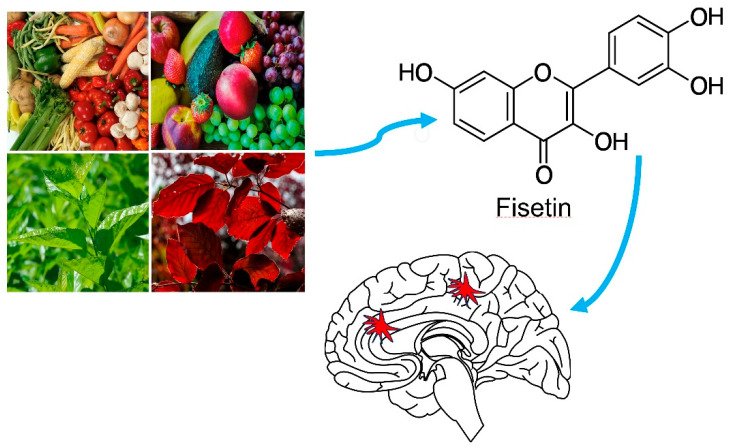
Overview of the natural sources of fisetin and its therapeutic potential in treating neurological disorders and health complications.

Fisetin belongs to a class of plant-derived flavonoids and is also the main component of the traditional Chinese medicine named mulberry leaves and Cotinus coggygria [7,8]. Apart from several fruits and vegetables, fisetin is abundantly present in various acacia trees and shrubs [6]. In addition, the flavonol bioactive molecule can be found in the Quebracho colorado and Rhus cotinus, lac tree, smoke tree, Pinopyta species like Callitropsis nootkatensis and other trees (Figure 1) [6,9]. Comparatively, the content of fisetin in strawberries is the highest (160 µg/g), while its content in apples (26.9 µg/g) and persimmons (10.5 µg/g) is relatively low [10]. Despite the low fisetin content in plants, it delivers immense health benefits with minimal adverse effects. This is mainly attributed to the natural synthesis of fisetin in various dietary plant resources [11]. The molecular formula of fisetin is C15H10O6, with a molecular weight of 286.2363 g/mol. Its melting point is 330 °C and its density is 1.688 g/mL. Fisetin has low lipophilicity (CLogP = 1.24) and a topological polar surface area of 107 Å. As to the solubility of fisetin, it is highly soluble (approximately 30 mg/mL) in dimethyl sulfoxide at 25 °C and appears yellow; in ethanol, its solubility is approximately 5 mg/mL; however, it is almost insoluble in water. Biochemically, fisetin has one rotatable bond with one covalently bonded unit count, six hydrogen bond acceptors and four hydrogen bond donors [6]. In the chemical structure of this molecule, fisetin has a diphenyl propane structure, which contains two aromatic rings connected by heterocycles oxidized through three carbon atoms, supplemented by four hydroxyl substituents and one oxygen group (Figure 1) [12]. This is a rare flavone without 5-hydroxy substitutions [6]. Biofunctionally, the bioactivity of fisetin predominantly relies upon the carbonyl group at four positions and with the double bond between C2 and C3, the OH groups at 3, 7, 3′, 4′ positions [9,13]. The OH groups at C3′, C3, and C-7, as well as the double bonds between C2 and C3, are linked to the antioxidant activity of fisetin [13]. In addition, due to its core structure consisting of 7-OH groups and 4-ketones, it has been proven to have anti-inflammatory properties [14]. In short, the specific physicochemical attributes of fisetin are implicated in a significant range of biological activities.

Although fisetin has a very low water solubility, which likely limits its bioavailability (44.1%) and therapeutic applications in clinical medicine, several lines of evidence indicate that the oral administration of fisetin is absorbed in the intestinal tract and passes the blood–brain barrier (BBB), reaching the CNS [15,16,17]. An in vitro study using MDR1-MDCK cells has demonstrated that fisetin has a high brain uptake potential [16]. In addition, an in vivo study demonstrated that orally administered fisetin in mice can disperse into the brain parenchyma [17]. These studies suggest that fisetin can be easily absorbed and enter the blood vessels. Likewise, another study also showed that after 40 min of oral administration, fisetin can be detected in the cerebral blood vessels for 2 h, indicating that fisetin is well-absorbed and bioavailable in the distal organs [17]. Moreover, apart from fisetin bioavailability and BBB permeability, fisetin can also affect hippocampal synaptic plasticity indirectly through the peripheral system [18]. Reportedly, the absolute bioavailability of fisetin was calculated as 7.8% and 31.7% after oral administration of fisetin at 100 and 200 mg/kg, respectively [19]. The main metabolites of fisetin are glucuronidated geraldol, geraldol and glucuronidated fisetin [20]. Whether orally (50 mg/kg of bw) or intravenously (10 mg/kg of bw), the parent form of fisetin exists for a short period of time, followed by the replacement of fisetin sulfates/glucuronides [21]. The oral administration of fisetin in animal studies revealed an effective dose range of 5–25 mg/kg, while its intraperitoneal doses range from 0.3 to 3 mg/kg when dissolved. In some clinical trials, the most commonly used oral dose is 20 mg/kg/day for two consecutive days and/or for two consecutive months [13,22]. Nevertheless, the efficient oral delivery of fisetin is still problematic due to its low aqueous solubility (less than 1.0 mg/mL), which impedes the oral bioavailability of fisetin [23,24]. To overcome the problem, the cocrystallization of fisetin with caffeine, isonicotinamide and nicotinamide was used to improve the solubility and oral bioavailability of fisetin [25,26]. Meanwhile, different formulation strategies have also been required for improvement of the bioavailability, such as nanoemulsion [27], liposome [24], and nanoparticles [28]. In addition, probiotics lactobacillus acidophilus cells were used as a new encapsulation carrier for the delivery of lipophilic fisetin [29]. There is also another option for improvement of fisetin bioaccessibility, namely the osmoporation technique to encapsulate fisetin using the probiotic bacteria Lactobacillus acidophilus [29]. A simulation study found that the hepatotoxicity and cytotoxicity of fisetin were not active [30]. Nevertheless, there is no evidence to suggest that fisetin has long-term or short-term toxicity.

Given that fisetin is commonly synthesized in plants such as fruits and vegetables, it is considered to be beneficial, effective and safe as a drug and dietary supplement. Based on these attributes and the extensive beneficial pharmacological effects, fisetin is becoming one of the prospective therapeutic agents and is accepted as a nutritional food or functional food to protect cells from various insults. For example, fisetin is currently marketed in the US by several nutriceutical companies either alone in 100 mg capsules or in combination with other natural products [20]. In addition, fisetin is used as a nutritional dietary supplement and a neuroprotective agent to prevent nervous system diseases. Until now, several preclinical studies have shown that fisetin has potential benefits against neurologic disease and neurological health complications [2]. In general, the indications for use include the improvement of cognition, maintenance of brain health and support of healthy aging [20]. It is estimated that the average human intake of fisetin for beneficial health is approximately 0.4 mg per day [12].

## 3. Neuroprotective Potentials of Fisetin

### 3.1. Fisetin and Neuroinflammation

It is well-known that a large number of neurological diseases, including AD, PD, HD, ALS, multiple sclerosis, brain cancer, encephalitis, epilepsy and others are multifactorial and heterogeneous in their pathogenesis [31]. Although these neurological diseases all manifest distinct pathological characteristics in different brain regions and have different etiologies, they are usually accompanied by a common mechanism such as neuroinflammation. Therefore, neuroinflammation is one of the important features involved in the progression of the abovementioned diseases. Generally, neuroinflammation is elicited by a variety of insults, which tends to be a secondary reaction triggered by early CNS injury (trauma, cancer, β-amyloid protein (Aβ) and hyperphosphorylated tau) and degeneration [32,33]. Notably, the core of common pathology of most neurological diseases is intimately associated with neuroinflammation whether the disease is acute or chronic. In the neuroinflammatory process, necrotic cells and tissues induced by pathogens can be eliminated [34]. Nevertheless, neuroinflammation is a double-edged sword that is not only required for recovery from a variety of diseases but also plays a harmful role in disease progress [31]. Consequently, the CNS inflammation is likely to be orchestrated by an interaction of microglial cells, infiltrating myeloid cells, astrocytes, oligodendrocytes, the BBB and signaling molecules (cytokines, chemokines, and growth factors) from the central and peripheral system [35].

#### 3.1.1. Microglia-Dependent Neuroinflammation in Nervous System Diseases

Microglial activation is an important hallmark of neuroinflammation and is seen in acute brain injury as well as in chronic neuroinflammatory and neurodegenerative diseases and plays a key role in regulating neuroinflammatory reactions [36]. Generally, microglial cells residing in the CNS play an important role in maintaining tissue homeostasis and contribute to neural development under physiological conditions. However, when the CNS suffers from various insults, microglia can be polarized into the M1 phenotype that secretes pro-inflammatory factors resulting in cytotoxicity, or the M2 phenotype that secretes anti-inflammatory or neuroprotective factors conducive to wound healing and tissue repair [37,38,39]. Notably, excessive microglial activation can worsen the surrounding healthy neural tissue, in turn resulting in the exacerbation of the chronic activation of microglia, as a result causing progressive loss of neurons [37]. Finally, the activated microglia lead to the spontaneous emergence of persistent inflammation by releasing a series of pro-inflammatory and neurotoxic factors, which further aggravate neurotoxicity and neurodegeneration [40].

Upon activation of microglia, the M1 phenotype microglia rapidly transform into an ameboid state, and migrate to the lesion site [36], resulting in the expression of pro-inflammatory mediators, including neurotoxic mediators (nitric oxide (NO), inducible nitric oxide synthase (iNOS), cyclooxygenase-2 (COX-2), prostaglandin E2 (PGE2)) and a series of pro-inflammatory cytokines (tumor necrosis factor α (TNF-α), interleukin 1β (IL-1β), interleukin 6 (IL-6), interleukin 8, interleukin 12, interleukin 23), CC chemokine ligand 2 and the suppressor of cytokine signalling-3 [32,41,42,43,44]. In addition, they can upregulate the expression of iNOS, causing the production of high levels of NO, nicotinamide adenine dinucleotide phosphate (NADPH) oxidase and radical oxygen species (ROS) [32,45]. All these harmful molecules further aggravate neuronal degeneration, leading to an uncontrolled degenerative cascade with concomitant expansion of the injury or neuropathic sites [33]. To date, many clinical trials have shown that the prevalence of CNS degenerative diseases was linked to inflammatory stimuli by increased cytokines as aforementioned and ROS during aging [46]. Additionally, M1 microglia express major histocompatibility complex-II, integrins (CD11b, CD11c), costimulatory molecules (CD36, CD45, CD47) and Fc receptors [43]. Overall, these molecules constitute an intricate microenvironment that is detrimental to neural regeneration. Conversely, the M2 phenotype microglia can be induced to produce anti-inflammatory cytokines such as IL-4, IL-10, IL-13, IL-10 and TGF-β. In addition, other growth-promoting factors released from the M2 phenotype like insulin-like growth factor-1, fibroblast growth factor, colony-stimulating factor 1 and neurotrophic growth factors [38,44] develop a microenvironment favorable to neural cell survival and regeneration. Furthermore, the M2 phenotype microglia also produce pro-survival factor progranulin and expressed mannose receptor (CD206), chitinase-3-like-3 and arginase 1 [43]. These secreted products play a pivotal role in the inhibition of pro-inflammatory responses and neuroregeneration. Therefore, it is critical to effectively inhibit M1 microglial polarization along with promoting M1 to M2 phenotypic conversion for the treatment of inflammation-related diseases.

#### 3.1.2. The Anti-Inflammatory Effect of Fisetin and Molecular Mechanism

**Figure 2:**
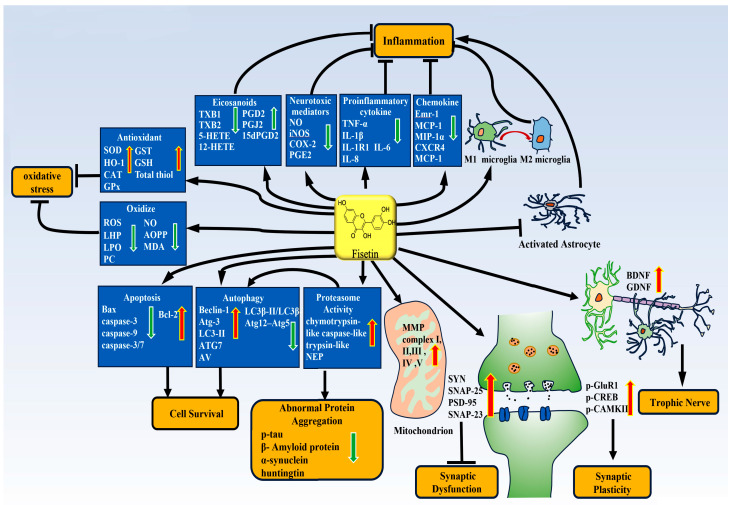
Schematic representation of multifaceted neuroprotective potentials of fisetin against diverse insults in neurological diseases. Fisetin is used to treat neurological diseases through a variety of mechanisms including anti-inflammatory, antioxidant stress, antiapoptosis, autophagy regulation, synaptic function improvement, enhancement of proteasome activity, improvement of mitochondrial function and neurotrophic effect. Uparrows (↑) and Downarrows (↓) represent upregulation and downregulation, respectively. Abbreviations: TXB1, thromboxane B1; TXB2, thromboxane B2; 5-HETE, 5-hydroxyeicosatetraenoic acid; 12-HETE, 12-hydroxyeicosatetraenoic acid; PGD2, prostaglandin D2; PGJ2, prostaglandin J2; 15d PGD2, 15-deoxy-PGD2; NO, nitric oxide; iNOS, inducible nitric oxide synthase; COX-2, cyclooxygenase-2; PGE2, prostaglandin E2; TNF-α, tumor necrosis factor α; IL-1 β, interleukin 1 β; IL-1R: interleukin-1 receptor; IL-6, Interleukin 6; IL-8, Interleukin 8; Emr-1, EGF-Like module-containing mucin-like hormone receptor 1; MIP-1α, microphage inflammatory protein-1α; CXCR4, chemokine (C-X-C motif) receptor 4; MCP-1, monocyte chemotactic protein-1; SOD, superoxide dismutase; HO-1, heme oxygenase-1; ROS, radical oxygen species; LPO, lipid peroxidation; LHP, lipid hydroperoxidation; PC, protein carbonyl; MDA, malondialdehyde; GSH, glutathione glutathione; CAT, Catalase; GST, Glutathione-S-transferase; GPx, glutathione peroxide; AOPP, Advanced oxidation protein products; AV, autophagic vesicles; SYN, Synaptophysin; SNAP-25, synaptosomal-associated protein 25; SNAP-23, synaptosomal-associated protein 23; PSD-95, postsynaptic density protein 95; CaMKII, Calcium–calmodulin (CaM)-dependent protein kinase II; CREB, cAMP response element-binding protein; NEP, neprilysin; MMP, mitochondrial membrane potential; BDNF, brain-derived neurotrophic factor; GDNF, glial-cell-derived neurotrophic factor.

Several studies suggested a potential anti-inflammation of fisetin in flavonoid-rich extract. Deng et al. found that in the rat model of sepsis-associated encephalopathy established by cecal ligation and puncture operation, fisetin (20 mg/kg) markedly attenuated neuroinflammatory responses by suppressing the expression of IL-1α, p-NF-κB, TNF-α and iNOS in rat microglia cells [7]. In addition, the activation of NLRP3 inflammasome was inhibited in cerebral microvascular endothelial cells, resulting in the decline of secretion of IL-1β into CNS, and thus, ameliorating cognitive impairment [7]. Similarly, another study showed that fisetin markedly suppressed the production of inflammatory mediators TNF-α, NO and PGE2 in lipopolysaccharide (LPS)-stimulated BV-2 cells or primary microglia cultures [47]. In addition, the expression of TNF-α, IL-1β, COX-2 and iNOS at both the mRNA and protein levels was also reduced [47]. Furthermore, it was reported that fisetin is nontoxic at 1–5 μM, dose-dependently reduced the production of iNOS and IL-1β and upregulated heme oxygenase-1(HO-1) levels [48]. In the ischemia/reperfusion model, fisetin (2–8 mg/kg) was identified as preventing cell damage and reducing the expression of inflammatory cytokines IL-1, TNF-α, iNOS, IL-1β, COX-2, IL-6 and PGE2 in a dose-dependent manner [49]. Other studies have also shown that fisetin (10–90 mg/kg) can inhibit the activation of astrocytes and microglia and then suppress inflammation in the brain of intracerebral hemorrhage mice by reducing the production of pro-inflammatory cytokines [50]. Strikingly, in a mouse model of vascular dementia by repeated ischemia-reperfusion of the total bilateral carotid artery, the delivery of fisetin (40 mg/kg) can alleviate the activation of inflammasome components NLRP-3, ASC and Caspase-1 and the release of their downstream products (IL-1β and IL-18), thus preventing apoptotic cell death [51]. In the model of stroke in vivo, pretreatment with 50 mg/kg fisetin sharply reduced the relative production of TNF-α from microglia [52]. Similarly, fisetin impeded the secretion of TNF-α in N9 cells in an in vitro inflammation model [52]. AlCl_3_-induced activation of microglia was inhibited by fisetin and cells exhibited drastic changes in morphology [53]. The IL-1R/TLR signal pathway is widely studied in the cascade reaction of neurites. It is reported that fisetin (10 and 20 mg/kg) can inhibit the expression of IL-1R1 and Toll-like receptor 4 (TLR4) in the hippocampus and cortex of epileptic mice [54], inhibit the migration of astrocytes and reduce the phosphorylation of focal adhesion kinase [55]. Furthermore, fisetin also inhibits cell proliferation by reducing the expression of cyclin D1 and arresting in G1 phase [55]. From these data, we speculated that fisetin plays a crucial role in suppressing neuroinflammation in neurological diseases by regulating glial cell phenotypes and the release of cytokines and chemokines (Figure 2). The upregulation of glial fibrillary acidic protein (GFAP) is a hallmark of astrocytic damage and often occurs in neuroinflammation, TBI, mental illness, brain edema-induced diseases, neurodegenerative disorders, etc [56]. In the mice model, fisetin treatment could effectively suppress the activation of astrocytes residing in the PM2.5-threatening cortex, hippocampus and hypothalamus, and 20 mg/kg fisetin can significantly reduce the expression of GFAP in these regions [57]. Meanwhile, the regulation of Emr-1, MCP-1, CD11b, MIP-1α, CXCR4 and Iba-1 was significantly suppressed [57], implying the anti-inflammation of fisetin. Interestingly, a fisetin-dependent reduction in GFAP levels was also seen in the hippocampus of the AD mice [58].

It is well known that eicosanoids play a key role in the regulation of inflammation. Fisetin could reduce the expression of COX-1, COX-2 and 12-lipoxygenase in AD mice, prevent the production of pro-inflammatory thromboxane B1 and thromboxane B2, and also increase the production of prostaglandin D2 (PGD2) and its non-enzymatic anti-inflammatory products prostaglandin J2 and 15-deoxy-PGD2 [58], as shown in Figure 2. In addition, fisetin decreases the levels of 5-hydroxyeicosatetraenoic acid and 12-hydroxyeicosatetraenoic acid, which are the main metabolites of 5-lipoxygenase and 12-lipoxygenase in AD mice [58]. Interestingly, the study also found that the levels of metabolic derivatives of various monohydroxy docosahexaenoic acids and docosahexaenoic acid could also be reduced by fisetin [58]. Collectively, these results suggest that fisetin emerges as an effective agent against inflammation-associated neurological diseases.

### 3.2. Fisetin and Antioxidative Stress

Oxidative stress is described as an imbalanced situation between the amounts of oxidants and their elimination through protective mechanisms such as antioxidants. When an imbalance occurs between antioxidants and reactive oxygen species (ROS), this will cause a mismatch between oxidative stress and antioxidant defense [59]. Once oxidant production is greater than antioxidant capacity, damage to important biomolecules such as nucleotides, proteins, proteins, carbohydrates and organs with potential impact on the whole organism is elicited. ROS, as a main oxidant molecule derivatives, include hydrogen peroxide, singlet oxygen, superoxide and hydroxyl radicals [60]. ROS can derive from various exogenous or endogenous sources. The exogenous production of ROS can be caused by chemicals, ultraviolet radiation and environmental toxins [61]. The endogenous production of ROS is mediated by mitochondrial and non-mitochondrial ROS-generating enzymes including flavin oxidases from peroxisomes, NADPH oxidase (Nox), endoplasmic reticulum cytochrome P450 and xanthine oxidase [61,62]. By contrast, the major ROS sources are the mitochondrial respiratory chain and the Nox system [63]. Most notably, the CNS is highly susceptible to oxidative stress, mainly owing to its narrow redox homeostatic window. The physiological concentration of ROS is essential for the preservation of cellular redox homeostasis as well as the regulation of cell proliferation and particular signaling pathways. However, the excessive production of ROS may result in a broad spectrum of cellular dysfunctions and adverse inflammatory changes, culminating in cell death. Sometimes, the neurotransmitters and excitatory amino acids act as a source of oxygen species like ROS, particularly present in the brain, causing oxidative stress damage [64]. ROS can induce protein oxidation and lipid peroxidation, leading ultimately to neuron death [33]. In addition, lipid peroxides and hydroperoxides strengthen the oxidative cascade by further generating relatively more stable and diffusible cytotoxic products such as malondialdehyde (MDA) [65]. Therefore, this cascade of oxidative damage further exacerbates the impact on the organism and tissues. Likewise, ROS and lipid peroxides have been implicated in the progressive pathogenesis of a variety of neurological insults, including epilepsy and neurodegenerative disorders [65]. Fortunately, glutathione (GSH) and GSH-associated metabolism provide the major line of defense for combating oxidative and other forms of toxic stress. GSH can eliminate electrophilic compounds and free radicals [66]. In addition, GSH also plays a crucial role in maintaining redox balance, regulating certain enzyme activities, cysteine transport/storage and cellular signaling [67]. Accordingly, the maintenance and generation of GSH by a novel antioxidant is likely to be an effective neuroprotective and therapeutic approach.

Oxidative stress is one of the most important pathogenic mechanisms for neurodegenerative diseases such as AD, PD, HD, stroke and TBI. Disease states lead to more production of ROS and subsequent oxidative damage. Some studies have shown that fisetin has direct antioxidant activity as demonstrated by a marked reduction in ROS production in cells and increased intracellular levels of GSH and the major endogenous antioxidant. This antioxidant activity is related to the structure, pH, and interactions with the cellular membrane of fisetin [60]. The synthesis of GSH depends on the activity of the rate-limiting enzyme, glutamate cysteine ligase (GCL) and the availability of cysteine (the precursor of the sulfur amino acid) [68]. Among them, GCL consists of modifier (GCLM) subunit and catalytic (GCLC) [68]. It is reported that fisetin can induce the expression of GCLC and GCLM [69]. More importantly, fisetin (10 μM) has been shown to prevent the reduction of two GCL subunits in primary rat neurons mediated by peroxynitrite donor SIN-1 [70]. This indicates that the antioxidant effect of fisetin is due to the enhancement of GSH synthesis, thus inhibiting the production of oxides. Notably, xCT is the cystine/glutamate antiporter solute carrier family 7 member 11, which promotes cystine uptake and GSH biosynthesis, thus protecting against oxidative stress [71]. The study showed that fisetin (5–20 µM) can mitigate tunicamycin-mediated PC12 cell death by scavenging ROS via overexpression of xCT [69]. These studies further revealed that fisetin is a multifunctional targeting agent for the suppression of oxidative stress.

For the treatment of neurological diseases, a study found that in the model of iron-induced epilepsy, pretreatment with fisetin (20 mg/kg) for 12 weeks reduced the production of MDA, a hallmark of the formation of lipid peroxides, and maintained the activity of Na^+^, K^+^-ATPase in the cerebral cortex and hippocampus of rats [65]. In addition, the treatment of amnesic mice with fisetin can significantly attenuate a decrease in GSH content and a decline of the activities of superoxide dismutase (SOD), glutathione reductase and glutathione peroxidase (GPx) in both the cortex and hippocampus of the scopolamine-treated mice [72]. Likewise, fisetin (10 and 50 mg/kg bw) was found to reduce the level of lipid peroxidation and protein carbonyl and preserve the levels of GSH and NO in the brain regions of pups belonging to methyl mercury (MeHg)-exposed mother rats [73]. Meantime, fisetin can increase the activity of SOD, catalase, GPx and glutathione-S-transferase, reduce the activity of glutathione reductase and preserve protein thiol and total thiol content of the brain regions [73], indicating fisetin’s antioxidative effects in the treatment of neurological diseases. Another interesting study on the TBI demonstrated that the administration of fisetin also markedly reduced the level of MDA and rebalanced the oxidation–antioxidation system [74], thus mitigating the development of neurotrauma. Fisetin also markedly upregulated hypoxia-inducible factor 1α, a kind of nuclear accumulation and hypoxia-response element (HRE)-driven transcriptional activator [75]. Similarly, the antioxidation of fisetin (15 mg/kg) by reducing the levels of ROS, lipid hydroperoxidation, protein carbonyl, intracellular calcium ion [Ca^2+^], NO and advanced oxidation protein products was also identified in a D-galactose-induced rat model [76]. These studies further implicate the protection of fisetin against various oxidation-induced cell degenerations in the brain, which is illustrated in Figure 2. Moreover, fisetin (10 μM) was found to suppress the 6-OHDA-mediated elevation of the oxidative-stress-related genes such as γ-glutamate-cysteine ligase modifier, NF-E2-related factor 2 (Nrf2), NAD(P)H: dehydrogenase quinone 1 and HO-1 [77], implying that fisetin’s antioxidative activity might play a crucial role in neuroprotection.

### 3.3. Fisetin and Autophagy

Autophagy is a vital lysosomal degradation and recycling pathway in eukaryotic cells responsible for maintaining cellular homeostasis, and necessary for neuronal survival and function [78]. Autophagy is the major cellular pathway to degrade dysfunctional organelles and protein aggregates. In the nervous system, neurons cannot dilute unwanted protein and organelle accumulation by cell division, therefore the nervous system is particularly dependent on autophagic pathways [79]. Abnormal autophagy has been implicated in the pathology of numerous diseases of the CNS, both chronic neurodegenerative disorders (such as AD, PD and HD) and many acute nerve injuries. Although autophagy is generally beneficial, its aberrant activation may also exert a detrimental role in neurological diseases depending on the environment and the insult, leading to autophagic neuronal death [80]. The administration of fisetin could upregulate the expression of autophagy genes Beclin-1 and Atg-3 in the brain of induced or naturally aged rats [76], implying fisetin’s neuroprotection against apoptotic cell death, neuroinflammation, and neurodegeneration in rat brain through autophagic pathways. Yang et al. showed that exposure to Pb led to injuries to the autophagy of neural cells, displaying increased expressions of LC3-II and Beclin-1 [81]. Intriguingly, fisetin (25 and 50 mg/kg) supplements increased these autophagy-related proteins and promoted the Pb-induced autophagy in the brains of mice [81]. This suggests that fisetin likely becomes a potential nutritional target for the prevention of Pb-induced neurotoxicity. Given that the conversion of LC3β from LC3β-I (free form, 18 kDa) to LC3β-II (phosphatidylethanolamine-conjugated form, 16 kDa) is an initiating step in autophagy in mammals [82], the decrease in LC3β-II/LC3β-I ratio and the formation of Atg12-Atg5 conjugation are critical for the formation of autophagosome [83]. It was shown that fisetin can reverse the increase in LC3β-II/LC3β-I ratio mediated by tunicamycin [69]. In addition, the formation of Atg12–Atg5 conjugation can be blocked by fisetin [69]. Nevertheless, it is noteworthy that fisetin could not only increase the amount of ATG proteins including Beclin-1 and ATG7 but also increase the number of autophagic vesicles in cells [84]. The detailed mechanism of the protective effect of fisetin against neurodegenerative insults needs to be further investigated.

### 3.4. Fisetin and Cyclin-Dependent Kinase 5 (Cdk5)

Cdk5 is a multifaceted serine/threonine protein kinase that is involved in regulating various physiological processes from neuron survival, migration and differentiation to synaptogenesis, synaptic plasticity and nerve transmission [85]. Cdk5 plays essential roles in a series of common nervous system diseases such as neurodegenerative diseases (AD, PD and frontotemporal dementia) [86], stroke, anxiety/depression, pathological pain and epilepsy [87]. However, under pathological conditions such as neurotoxic damage and neurodegenerative diseases, Cdk5 can be aberrantly activated and lead to the initiation and progression of various neurological diseases by affecting downstream targets such as mitochondrial fragmentation in cerebral ischemia, apoptosis of dopaminergic neurons in PD and Aβ protein formation in AD [88]. Notably, normal Cdk5 activity is conferred mainly by association with its primary activator p35 [89]. Under neurotoxic conditions, p35 undergoes proteolytic cleavage to p25, which implicates various neurodegenerative diseases. Thereafter, p25 becomes a potent and mislocalized hyperactivator of Cdk5, resulting in a deregulation of Cdk5 activity [89]. The accumulation of p25 is considered to be the pathogenic factor of AD, and the overexpression of p25 in vivo leads to the formation of phosphorylated tau, neurofibrillary tangles and cognitive defects, which are all pathological signs of AD [89]. Similarly, several studies have indicated that fisetin reduces the level of Cdk5, activator p35 cleavage product p25 in the brain of APPswe/PS1dE9 transgenic AD mice [58,90,91], thereby leading to the inhibition of neurodegeneration [58].

### 3.5. Fisetin and Apoptosis

Apoptosis, also named programmed and orchestrated cell death, is a highly conserved cellular process. Apoptosis plays a vital role in a wide variety of pathological and physiological processes such as maintaining balance in the body, tissues and organs development, cellular degeneration and immune responses [92]. The mechanism of apoptosis is complex and involves many pathways. Apoptosis is initiated by microenvironmental perturbations that result in initiator caspases (caspase-8 or -9) activating downstream executioner caspases (caspase-3, -6 and -7) [93]. In addition, the antiapoptotic Bcl-family regulator proteins and executors of intrinsic proapoptosis and modulators Bax and p53 play a pivotal role in the occurrence of apoptosis events. Therefore, the regulation of the underlying endogenous protective signaling cascades may be useful for the prevention and treatment of neurological disorders. It was reported that fisetin significantly lowered the ratio of the proapoptotic Bax protein and the antiapoptotic Bcl-2 protein in SH-SY5Y cells [77]. In addition, fisetin effectively suppressed the 6-OHDA-mediated activation of Caspase-3 and Caspase-9, which results in cell death. Meanwhile, 6-OHDA-induced caspase-3/7 activity was attenuated [77], indicating the neuroprotective roles of fisetin against 6-OHDA-induced apoptosis. Consistently, it has also been found that fisetin could reverse the increase in Bax and Caspase-3 expression and the Pb-induced decrease in Bcl-2 expression in mouse brain [81]. Similarly, Zhang et al. showed that fisetin effectively increased the expression of Bcl-2 while decreasing the expression of Bax and Caspase-3 after TBI, preventing neuronal cell death and apoptosis [74]. Moreover, several lines of studies demonstrated that fisetin reduces Aβ aggregation, ASK-1, p-JNK, p53, cytochrome C, Caspase-9 and -3 protein expressions and modulates Bax/Bcl-2 ratio in AlCl_3_-induced rats [94]. Nevertheless, in the glioma, fisetin upregulates the expression of Caspase-3, Caspase-9, Caspase-8 and Bax by the selected doses of 25 and 50 μM, while that of Bcl-2 and survivin was reduced in T98G cells [95]. These results implicated that fisetin plays a wide range of roles in suppressing neural cell death and promoting tumor necrosis through the apoptosis pathway.

### 3.6. Fisetin Improves Synaptic Function

Synapses are specialized structures that mediate information flow between neurons and target cells and thus. are the basis for the neuronal system to execute various functions. After the establishment of synaptic connections, synapses constantly undergo structural or functional changes, known as synaptic plasticity which is the capacity of a synapse to adapt to overall neuronal activity. In general, many nervous system diseases are associated with the dysfunction of synapses. The disruption of synaptic proteins including synaptosomal-associated protein 25 (SNAP-25), postsynaptic density protein 95 (PSD-95), cAMP response element-binding protein (CREB), Calcium–calmodulin (CaM)-dependent protein kinase II (CaMKII) and others will inevitably cause synaptic dysfunction because they serve a crucial role in synaptic plasticity [81]. Reportedly, presynaptic protein SNAP-25 and postsynaptic protein PSD-95 are essential for brain development, synaptic connection, neuronal maturation and plasticity [96,97]. Accordingly, the maintenance of these synaptic protein activities is crucial for numerous neural functions. Strikingly, there is mounting evidence that fisetin (20 mg/kg) treatment markedly reverses Aβ1-42-induced synaptic dysfunction by increasing the levels of both presynaptic (SYN and SNAP-25) and postsynaptic proteins (PSD-95, SNAP-23, p-GluR1, p-CREB and p-CAMKII) and ultimately, improves animal neurologic deficits, as observed in the Morris water maze test [97]. Coincident with this, it has been reported that fisetin could increase the expression of PSD-95, SNAP-25, p-CREB and p-CaMKII in the brain of Pb-treated mice, indicating that fisetin can alleviate the Pb-caused impairment of learning and memory by improving the synaptic function [81]. In addition, fisetin can also restore the levels of PSD-95 phosphorylation and PSD-95-related protein drebrin in AD brain [58]. It is well-known that synaptic acetylcholinesterase (AChE) terminates transmission at cholinergic synapses by rapidly hydrolyzing acetylcholine [98]. A rapid removal of free acetylcholine from the synapse is essential for non-cholinergic effects such as normal muscle function, learning, cognitive memory, control of cortical tissue and cerebral blood flow of movement, cell proliferation and neurite growth [98]. Another study demonstrated that fisetin reduced the level of AChE in the cortex and hippocampus, indicating the regulatory role of fisetin in cholinergic functioning, thereby regulating oxidative stress and memory deficits [53]. In addition, fisetin was found to reverse the increase in brain AChE activity caused by aging [76] and reduce the increase in brain AChE activity induced by DL-Hcy in rats [99]. Notably, synaptophysin, a transmembrane glycoprotein, exists in presynaptic vesicles of nerve cells and vesicles of neuroendocrine cells [100] and plays an important role in synaptic plasticity through the phosphorylation and release of neurotransmitters [101]. Inspiringly, it was found that fisetin can reverse the repressed synaptophysin and Gria1 genes, and increase the phosphorylation and surface expression of AMPAR GluA1 subunit in rats [102,103]. Consistent with this finding, fisetin (30 mg/kg) also can regulate the expression of genes and proteins involved in synaptic transmission and plasticity, and prevent the downregulation of neurogranin, dendritic protein, synaptic fusion protein 1A, Lin-7 homolog A, Complexin-2 and Exolyst complex component 8 induced by MeHg exposure [104]. Importantly, fisetin could prevent the decrease in three proteins linked with synaptic functions such as activity-regulated cytoskeleton-associated protein, Homer, and synapse-associated protein 102, in aged mice [105]. Based on these results on fisetin as a potential therapeutic for AD and other age-related neurodegenerative diseases as shown in Figure 2, we speculate that maintenance of the levels of these proteins involved in synaptic function is greatly attributed to the positive modulation by fisetin.

### 3.7. Fisetin and Proteasome Activity and Abnormal Protein Aggregation

Neuronal death in neurodegenerative diseases is usually associated with misfolded proteins that aggregate within the brain as a result of disease linked to gene mutations or abnormal protein homeostasis. However, aging is generally accompanied by strong metabolic alterations in a variety of aspects including proteasome degradation and autophagy decrease, followed by Aβ and tau α-increase in potential neurotoxic protein aggregates, loss of synaptic nucleoprotein, SOD1 and TDP-43 [106]. The ubiquitin–proteasome system and the autophagy–lysosome pathway are two main degradation pathways that can eliminate unwanted or misfolded proteins in cells to prevent their accumulation and maintain cell health [106]. For example, a previous study demonstrated that mutant huntingtin-expressing cells significantly decreased chymotrypsin-like and caspase-like activities [107]. In addition to the activities of chymotrypsin-like and caspase-like, the activity of trypsin-like was also reduced in the white matter and gray matter of patients with multiple sclerosis [108], suggesting the importance of the levels of these protein alterations in neurodegenerative diseases. Interestingly, fisetin was shown to modestly increase the chymotrypsin-like activity of the proteasome in primary cortical neurons [109]. In further studies with the HT22 nerve cell line, fisetin was also found to increase the activities of these proteasomes in a time- and dose-dependent manner [16]. Aβ aggregation and high levels of hyperphosphorylated tau protein are common hallmarks of AD [110]. Neprilysin (NEP) is a major endogenous catabolic enzyme of Aβ [111]. Thereby the increase in NEP expression ameliorates the symptoms of AD [111]. Critically, fisetin can reduce the deposition of phosphorylated-tau and Aβ and increase the expression of NEP in the brain [81]. More significantly, fisetin also inhibits the aggregation of tau fragment K18 and disaggregates tau K18 filaments [112]. The biological activity of fisetin is exerted by preventing the formation of β-strands and its binding to tau K18 protein [112]. The degradation of AChE, β-site amyloid precursor protein cleaving enzyme 1 (BACE1), as well as the interaction between Aβ and amyloid-binding alcohol dehydrogenase (ABAD), are key factors that exacerbate the pathological process of AD [113]. Therefore, the use of fisetin to interfere with the binding ability of these proteins is likely to be effective for the treatment of AD. Interestingly, significant binding and acceptable binding values were found when using two different tools, FlexX and iGEMDOCK, to perform docking experiments between fisetin and these proteins [113]. The neuropathological features of PD are the progressive loss of dopamine neurons in the midbrain and the formation of aggregates composed mainly of a-synuclein protein [114]. It is reported that fisetin can reduce the cytotoxicity and cell death induced by MPTP/MPP+ by decreasing α-synuclein expression [115]. Remarkably, fisetin could reduce the percentage of cells containing α-synuclein inclusions as well as their size and subcellular localization in a yeast model of α-synuclein aggregation [116]. Likewise, fisetin can combine with α-Syn through hydrophobic interaction and increase the stability of α-Syn, resulting in an increase in the inhibitory ratio of fisetin to α-Syn fibrillation [117]. Huntington protein is a well-known mutant protein in HD. Mutated Huntington protein interferes with many key cellular functions, such as mitochondrial function, transcription, and protein translation, ultimately leading to cell death [118]. Reversely, treatment of PC12 cells with fisetin effectively inhibited the expression of mutant Huntington protein, leading to an increase in cell viability [119]. Furthermore, this study also demonstrated that fisetin inhibited the direct toxicity of the mutant protein. In light of current data, fisetin exerts neuroprotection against neural degeneration by modulation of proteasome activity and abnormal protein aggregation, as shown in Figure 2.

### 3.8. Fisetin and Mitochondrial Function

Mitochondria play a critical role in cell life and cell death. However, dysfunction of mitochondria indicates a variety of pathological processes and the occurrence of diseases. Mitochondrial dysfunction is thereby becoming one of the most emerging pathological processes in the etiology of various diseases including neurological disorders. Based on this, the protection of mitochondrial function against a spectrum of insults has been identified as the most effective therapeutic strategy to attenuate the pathogenesis of neurodegenerative diseases. Due to the loss of mitochondrial membrane potential responsible for its permeability, the impairment of mitochondrial membrane permeability is the first step for the onset of apoptotic events [76]. It was shown that fisetin reduces the aging-induced loss of mitochondrial membrane potential by reducing the mitochondrial membrane depolarization of brain cells in naturally aged rats [76]. Most importantly, Alikatte et al. found that fisetin increases mitochondrial enzyme activity [120], indicating fisetin-induced protection of mitochondrial function. Similarly, oral feeding of fisetin results in the restoration of brain NADH-dehydrogenase, brain mitochondrial SDH level, brain mitochondrial MTT (Complex-III) and brain mitochondrial cytochrome oxidase (Complex-IV) in rats with chronic mild hyperhomocysteinemia [99]. Moreover, studies have demonstrated that oral administration of fisetin (30 mg/kg) significantly reversed MeHg-induced mitochondrial swelling and the decrease in mitochondrial ETC [complex I, II, IV and V] activity in the rat hippocampus [121]. These studies clearly demonstrated that fisetin has the potential to protect mitochondrial functions against various insults, enhancing the survival of nerve cells and attenuating of development of a variety of diseases.

### 3.9. Fisetin and Neurotrophic Effects

Neurotrophic factors are multifunctional signaling proteins that regulate and promote the development, maintenance and survival of neurons and neuronal supporting cells such as glial cells and oligodendrocytes. Until now, a large amount of evidence showed that the level of neurotrophic factors or their receptors are intimately neuronal death and aging, as well as the pathogenesis of abnormal neurotrophic support (such as neurodegenerative diseases and depression) and excitation (such as epilepsy and central pain sensitization) [122,123]. Treatment with fisetin greatly upregulated the expression of brain-derived neurotrophic factor (BDNF) and glial-cell-derived neurotrophic factor (GDNF) in high glucose-treated HT22 cells [102]. Furthermore, treatment of fisetin prevented MeHg-induced downregulation of the levels of BDNF and GDNF expressions and hampered reactive astrogliosis in the hippocampus of F1 generation rats [104]. The data of these studies are indicative of fisetin-induced nourishing of the neurons by regulating neurotrophic factors.

### 3.10. Fisetin and Target of Sirtuins

Sirtuins 1–7, a class of NAD^+^-dependent deacylases, belong to class III histone deacetylase enzymes. The enzymatic activity of sirtuins is linked to gene repression, metabolic control, apoptosis and cell survival, DNA repair, development, inflammation, neuroprotection and healthy aging [124]. Although sirtuins are initially identified as deacetylases, they are currently confirmed to have much more kinds of enzymatic activity, including deacylase and O-ADP-ribosylase activity. Recent studies showed that sirtuins enhance synaptic plasticity, influence epigenetic regulation and play key roles in molecular pathways involved with oxidative stress affecting mitochondrial function [125]. Although sirtuins 1–7 share a similar catalytic domain and use NAD^+^ as a co-substrate, they possess different profiles of actions, substrate affinity and subcellular compartmentation. In the CNS, sirtuin-1 promotes axon elongation, neurite growth and dendritic branching, and also plays a role in memory formation by regulating synaptic plasticity [126]. Sirtuin-2 mainly exists in the cytoplasm of the CNS, which is generally found to accelerate the development of neuropathology and protect the brain from deterioration in some cases [127]. A recent study demonstrates that fisetin decreases the expression of the sirtuin-2 gene in aging mice and increases the level of sirtuin-1 [76], implying the neuroprotective potential of fisetin via the modulation of sirtuins. Similarly, neurochemical examination indicated that fisetin administration reversed the decrease in SIRT1 expression in tunicamycin (1 µg/mL)-treated PC12 cells [69]. These results indicate that fisetin significantly suppresses neuronal toxicity, possibly via modulating sirtuin-1 activation and expression.

**Table 1:** Fisetin exerts neuroprotective effects through multiple molecular mechanisms.

Effects of Fisetin	Suggested Mechanism	References
Anti-inflammation	Suppress inflammatory factors and chemokine such as TNF-α, IL-1β, IL-6, IL-8, iNOS, COX-2, PGE2, IL-18, Emr-1, MCP-1m, MIP-1α, CXCR4, MCP-1	[49,51,54,57,76]
	Reduce the expression of TXB1, TXB2, 5-HETE, 12-HETE and increase the expression of PGD2, PGJ2, 15dPGD2	[58]
	Reduce the polarization of M1 phenotype and production of inflammatory mediators in microglia	[7,47,52,53,72]
	Inhibit the activation of astrocytes and reduce the expression of GFAP	[53,57]
	Reduce the level of p25	[58]
	NLRP-3, ASC and cleaved-caspase-1 ↓	[51]
	NF-κB activation ↓	[51,52,57]
	TLR4/MyD88/NF-κB ↓	[81]
	Phosphorylation of JNK ↓	[52]
	Phosphorylation of ERK ↓	[72]
	IL-1R/TLR Axis ↓	[54]
Antioxidative stress	Reduce oxide production such as ROS, LHP, LPO, PC, NO, AOPP, MDA	[60,65,69,73,74,75,76]
	Increase the production of antioxidants such as SOD, HO-1, CAT, GPx, GSH, GST, Total thiol	[60,69,70,72,73,74,76]
	Nrf2-ARE ↑	[51,74]
	HIF-1α/HRE ↑; phosphorylation of MEK1/2 and p38 MAPK; ↑ PI3K/Akt ↑	[75]
	Hyperphosphorylation of ERK and phosphorylation of c-Myc ↓; Nrf2 ↑	[70]
	Phosphorylation of ERK, JNK (c-JUN NH2-terminal protein kinase), and p38 MAPK ↑; SIRT1 ↑; Nrf2 ↑	[69]
Regulation of autophagy	Upregulation of autophagy genes (Atg-3 and Beclin-1)	[76]
	Increase these autophagy-related proteins LC3-II and Beclin-1	[76]
	Increase the number of autophagic vesicles and ATG proteins including Beclin-1 and ATG7	[84]
	Stimulate autophagic degradation of phosphorylated tau via the activation of TFEB and Nrf2 transcription factors ↑	[84]
	Reduce the ratio of LC3β-II/LC3β-I and the formation of Atg12-Atg5 conjugation	[69]
	mTORC1 ↓	[84]
Antiapoptosis	Reduce the expression of proapoptotic proteins such as BAX, caspase-3, caspase-9 and caspase-3/7	[74,77,81,94,95]
	Increased the expression of antiapoptotic proteins such as Bcl-2	[16,81,109,112,115,116,117,119]
	PI3K-Akt ↑	[77]
Increase in the activity of proteasomes and reduction in the aggregation of abnormal proteins	Increase the chymotrypsin-like activity of the proteasome	[16,109]
	Increase the expression of NEP in the brain and reduce the deposition of phosphorylated-tau	[81]
	Decrease α-synuclein expression	[115]
	Reduce the percentage of cells containing α-synuclein inclusions as well as their size and subcellular localization	[116]
	Increase the inhibitory ratio toα-synuclein fibrillation	[117]
	Inhibit the expression of mutant Huntington protein	[119]
	Activation of Ras-ERK ↑	[119]
Improvement of mitochondrial function	Reduce loss of mitochondrial membrane potential	[76]
	Increase mitochondrial enzyme activity	[120]
	Restore the level of brain NADH-dehydrogenase, brain mitochondrial SDH level, brain mitochondrial MTT (Complex-III), and brain mitochondrial cytochrome oxidase (Complex-IV)	[99]
	Reverse MeHg-induced mitochondrial swelling and decrease in mitochondrial ETC (complex I, II, IV & V) activity	[121]
Improvement of synaptic function	Increase the levels of both presynaptic (SYN and SNAP-25) and postsynaptic proteins (PSD-95, SNAP-23, p-GluR1, p-CREB and p-CAMKII)	[81,97]
	Restore the levels of PSD-95 phosphorylation and PSD-95-related protein drebrin	[58]
	Reverse the increase in brain AChE activity	[76,99]
	Reverse the repressed synaptophysin and Gria1 genes and increase the phosphorylation and surface expression of AMPAR GluA1 subunit	[102,103]
	Regulate the expression of genes and proteins involved in synaptic transmission and plasticity and prevent the downregulation of neurogranin, dendritic protein, synaptic fusion protein 1A, Lin-7 homolog A, Complexin-2 and Exolyst complex component 8	[104]
	Prevent the decrease in three proteins linked with synaptic functions such as activity-regulated cytoskeleton-associated protein, Homer, and synapse-associated protein 102	[105]
Improvement of memory and cognition	Upregulate expression of neurotrophic factors such as BDNF and GDNF	[102,104]
	Increased expression of synaptic proteins and improved synaptic function	[97,102,103,104,105]
	Reduce the level of AChE	[53,76,99]
	Activation of Ras-ERK ↑	[119]
	Phosphorylation of ERK/CREB ↑	[15,103]
	Activation of PI3K/Akt/CREB ↑	[102]

Uparrows (↑) and Downarrows (↓) represent increase and decrease, respectively.TNF-α, tumor necrosis factor α; IL-1β: interleukin 1β; IL-6: Interleukin 6; IL-8: Interleukin 8; IL-18: Interleukin 18; iNOS, inducible nitric oxide synthase; COX-2, cyclooxygenase-2; PGE2, prostaglandin E2; Emr-1, EGF-Like module-containing mucin-like hormone receptor 1; MIP-1α, microphage inflammatory protein-1α; CXCR4, chemokine (C-X-C motif) receptor 4; MCP-1, monocyte chemotactic protein-1; TXB1, thromboxane B1; TXB2, thromboxane B2; 5-HETE, 5-hydroxyeicosatetraenoic acid; 12-HETE, 12-hydroxyeicosatetraenoic acid; PGD2, prostaglandin D2; PGJ2, prostaglandin J2; 15d PGD2, 15-deoxy-PGD2; MeHg: methyl mercury; GFAP: glial fibrillary acidic protein; NLRP-3: NOD-like receptor thermal protein domain associated protein 3; NF-κB: nuclear factor kappa B; TLR4: Toll-like receptor 4; MyD88: Myeloid differentiation primary response gene 88; ERK: extracellular signal-regulated kinase; JNK: c-Jun N-terminal kinase; TFEB: Transcription factor EB; Nrf2: NF-E2 related factor 2; mTOR: mechanistic target of rapamycin; PI3K: Phosphoinositide 3-kinase; NEP, neprilysin; AChE: acetylcholinesterase; CREB: cAMP response element-binding protein.

## 4. Signal Pathway

### 4.1. Fisetin and NF-κB Pathway

**Figure 3:**
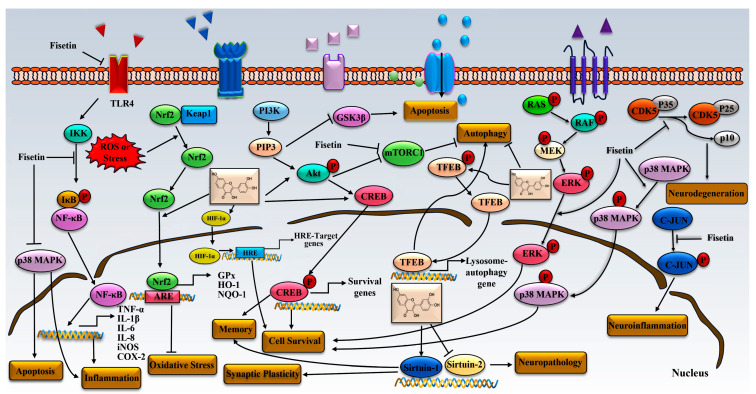
Schematic diagram of the potential signaling pathways underlying the neuroprotective effects of fisetin against neurological disease. The potential of fisetin has been highlighted in the modulation of different neuroprotection-related signaling pathways, mainly including NF-κB, Keap1/Nrf2/ARE, PI3K-Akt, MAPK and TFEB, which are associated with the initiation and progression of neurological diseases. Abbreviations:TLR4: Toll-like receptor 4; NF-κB: nuclear factor kappa B; IKK: IκB kinases; ROS: radical oxygen species; iNOS: inducible nitric oxide synthase; COX-2: cyclooxygenase-2; TNF-α: tumor necrosis factor α; IL-1β: interleukin 1β; IL-6: Interleukin 6; IL-8: Interleukin 8; GPx: glutathione peroxide; HO-1: heme oxygenase-1; NQO1: NAD(P)H: quinine oxidoreductase 1; Nrf2: NF-E2-related factor 2; Keap1: Kelch-like ECH-associated protein 1; ARE: antioxidant-response element; HIF-1α: hypoxia-inducible factor 1α; HRE: hypoxia-response element; PI3K: Phosphoinositide 3-kinase; PIP3: Phosphatidylinositol Triphosphate; GSK3β: Glycogen synthase kinase-3 beta; CREB: cAMP response element-binding protein; mTOR: mechanistic target of rapamycin; CDK5: Cyclin-dependent kinase; TFEB: Transcription factor EB; RAS: Reliability, availability and serviceability; ERK: extracellular signal-regulated kinase; MAPK: mitogen-activated protein kinase.

The nuclear factor kappa B (NF-κB) is a transcription factor and a key mediator of inflammatory response. It mainly causes inflammation by regulating the expression of various pro-inflammatory genes, including adhesion molecules, chemokines and cytokines [128]. In general, there are two different pathways for NF-κB signaling: the noncanonical and canonical. The canonical activation of NF-κB is mainly due to the phosphorylation of the IκB kinases (IKKs) complex, which leads to the phosphorylation of IkappaB and its ubiquitination–degradation by proteasome, thereby causing NF-kappaB to translocate to the nucleus and activate the target gene [129,130]. NF-κB entering the nucleus binds to the DNA of chemokines, pro-inflammatory cytokines and other proteins such as iNOS and COX-2 [131], resulting in their expression. Upon in vitro experimental evaluation of a variety of flavonoids using a well-studied intracerebral hemorrhage mice model, fisetin (90 mg/kg) was shown to attenuate NF-κB activation, as demonstrated by increased cytoplasmic p-65, IκBα, and reduced cytoplasmic p-IκBα, nucleus p-65 [50]. In addition, some studies have demonstrated that after LPS stimulation of microglia, fisetin (0.5–2 µg/mL) suppressed the translocation of p65 into the nucleus and degradation of IκB in a dose-dependent manner [47]. Similarly, the NF-κB signaling pathway was identified to display an important role in promoting neuroinflammatory responses in activated astrocytes of PM2.5-induced mice [57]. Interestingly, the study found that fisetin exerts an anti-inflammatory effect in a dose-dependent manner by inhibition of NF-κB signaling [57]. Furthermore, fisetin (25 and 50 mg/kg) could improve neurological function by suppressing LPS-induced inflammation mediated by the TLR4/NF-κB pathways [132]. In addition, the administration of fisetin (25 and 50 mg/kg) can inhibit the activation of the TLR4/MyD88/NF-κB signaling pathway by Pb and thus, decrease the expression of pro-inflammatory mediators IL-6 and TNF-α [81]. Together, fisetin exerts the neuroprotective effect against inflammatory insults in the CNS through the regulation of neuroinflammatory responses via TLR4, MyD88 and NF-κB as shown in Figure 3, suggesting fisetin might be a promising anti-inflammatory agent for neurodegeneration and nerve injury.

### 4.2. Fisetin and Keap1/Nrf2/ARE Pathway

The Nrf2 and antioxidant-response element (ARE) pathway is a key regulator for modulating inflammation and oxidative damage. Kelch-like ECH-associated protein 1(Keap1), a cytoskeletal actin-binding protein, is an inhibitor of Nrf2 that sequesters it in the cytoplasm. Under physiological conditions, Nrf2 usually forms a complex, resulting in Nrf2 inactivation via ubiquitination and degradation [133]. However, under oxidative and xenobiotic stress conditions, Nrf2 dissociates from Keap1, and subsequently translocates to the nucleus, resulting in the activation of numerous antioxidant enzymes such as GPx, HO-1 and nicotinamide adenine dinucleotide phosphate, quinine oxidoreductase-1 (NQO-1) via interaction with the ARE [134]. The present study indicated that the Keap1/Nrf2/ARE signaling pathway mediates the preventive and protective effects on CNS diseases, including stroke and TBI, and neurodegenerative diseases such as AD and PD [133]. Previous studies suggested the Keap1-Nrf2 complex as a main molecular target of fisetin in vivo [74]. It was reported that fisetin inhibited ubiquitination-mediated Nrf2 degradation, leading to the translocation of Nrf2 from the cytoplasm to the nucleus [135]. Subsequently, fisetin activates Nrf2-ARE, resulting in the expression of downstream phase II enzymes, such as glutathione-S-transferase, NAD(P)H: dehydrogenase quinone 1, HO-1, GCLC and GCLM. The phase II enzymes scavenge excessive oxidants and their derivatives in cells to counteract intracellular oxidative damage [135]. Similarly, another study showed that fisetin effectively promoted the translocation of Nrf2 into the nucleus, and enhanced the binding ability of Nrf2 to ARE [74]. Collectively, these studies provided substantial evidence that fisetin plays a critical role in neuroprotection partly through the activation of the Keap1/Nrf2/ARE pathway.

### 4.3. Fisetin and PI3K-Akt

Phosphoinositide 3-kinase (PI3K), a member of the lipid kinase family, can be activated by multiple signalings and involved in the regulation of multiple signaling pathways by interacting with BTK, PDK1 and Ra multiple signaling molecules. Akt is a serine/threonine kinase that belongs to the AGC kinase family and can link the interaction between receptors and PI3K, which results in numerous cellular events. In the CNS, Akt has been reported to regulate neuronal toxicity through its various substrates like FOXos, GSK3β, caspase-9, etc. [136]. The PI3K/Akt pathway is unique for its multitude of roles in various cellular processes, including cell proliferation, apoptosis, cell migration, vesicle transport, angiogenesis and cell cancerous transformation [137]. It was found that fisetin reversed 6-OHDA-induced cell death by activating the PI3K/Akt signal pathway in human neuronal SH-SY5Y cells, and this effect can be offset by co-treatment of Akt inhibitors [77]. In addition, fisetin can enhance the phosphorylation of CREB, Akt and PI3K in HT22 cells induced by high glucose to enhance the neuroprotective effect [75,102]. Furthermore, fisetin effectively inhibits astrocyte proliferation, and further reverses glial scar formation by suppressing the phosphorylation level of Akt and extracellular signal-regulated kinase (ERK) in astrocytes in a dose-dependent manner [55]. The mechanistic target of rapamycin (mTOR) plays a crucial regulatory role in cell growth and metabolism. Interestingly, it was found that fisetin significantly upregulated the ratio of phosphorylated Akt/total Akt and phosphorylated mTOR/total mTOR in both the hippocampus and cortex of pentylenetetrazole-kindled mice [54]. The results suggest that the PI3K/Akt signal pathway actively contributes to fisetin-mediated suppression of apoptotic molecules in the hippocampus and cortex.

### 4.4. Fisetin and MAPK Pathway

The MAPK family members (p38, JNK and ERK) play prominent roles in regulating multiple cellular processes, such as development, stress response, apoptosis, transformation, differentiation and proliferation [138]. The activation of MAPK in neural cells is influenced by various external stimuli, such as cellular stress, glutamate and hormones, pathogens and growth factors [139]. Therefore, the upregulation of p-MAPK expression usually implicates intracellular activities via the sequential phosphorylation of substrate protein Ser/Thr kinase protein cascades. Recent studies showed that fisetin regulates the activation of ERK, JNK and p38 MAPK [69]. ERK is a kinase that regulates the proliferation, differentiation and survival of nerve cells [136]. Fisetin exerts antioxidant and neuroprotective effects in multiple mutant human SOD1 models of ALS by activating ERK [140]. The activation of the ERK pathway likely contributes to the expression of antioxidant protein and the protection against ROS-induced nervous system injury [140]. In addition, fisetin increases the levels of p-ERK and HO-1, and decreases the level of human SOD1 [140]. Fisetin also induces CREB phosphorylation in rat hippocampal slices, facilitates long-term potentiation in rat hippocampal slices and enhances object recognition memory in mice [15]. More importantly, other impaired behaviors were reversed in AD mice fed fisetin [15]. CREB is a nuclear transcription factor essential for memory formation, neuronal plasticity and the apoptosis of hippocampal neurons [141]. In a detailed study, fisetin was demonstrated to improve memory impairment and other neurological diseases including AD and PD by increasing p-CREB [97]. Moreover, fisetin can protect against both GSH decrease and cell death by inhibiting ERK hyperphosphorylation and c-Myc phosphorylation, as well as preventing a decrease in Nrf2 [70]. p38 MAPK is highly expressed in the different regions of the CNS that are tightly associated with learning and memory, and now emerges as a key player in synaptic regulation and function [142]. Apart from this, p38 MAPK also actively contributes to neuroinflammation caused by glial cells, including microglia and astroglia. More notably, it is reported that fisetin could save the viability of PC12 cells under hypoxia conditions through upregulation of phospho-ERK1/2 and phospo-p38 [75]. p-JNK is the main hallmark responsible for neuroinflammation and neurodegeneration [115]. More than that, fisetin was demonstrated to reduce inflammation by suppressing the phosphorylation of JNK and c-Jun [52,115]. These effects were the results of fisetin-induced phosphorylation of the ERK, JNK and c-Jun proteins in cellular response to a diverse array of stimuli.

### 4.5. Fisetin and TFEB

Transcription factor EB (TFEB) is considered to be the main transcriptional regulator of autophagy, lysosomal exocytosis and lysosomal biogenesis. Under normal conditions, TFEB is located in the cytoplasm and remains on the surface of lysosomes, and its activity and subcellular localization are influenced by rapamycin complex 1 (mTORC1)-mediated phosphorylation [143]. When subjected to certain stimuli, TFEB undergoes dephosphorylation and translocation to the nucleus to induce target gene transcription [143]. TFEB has attracted much attention due to its ability to induce intracellular clearance of pathogenic factors in a variety of disease models. Therefore, seeking TFEB agonists is a promising strategy for the treatment of diseases related to autophagy dysfunction. It has been reported that fisetin can increase the mRNA level of TFEB downstream genes such as ATG9b and LAMP1 in cortical cells and primary neurons [84]. In addition, fisetin (2.5–10 μM) reduces the phosphorylation levels of the downstream protein 4E-BP1 and p70S6 kinase of mTORC1 in cortical cells in a concentration-dependent manner [84]. Therefore, the activation of TFEB and autophagy induced by fisetin is likely attributed to its ability to directly inhibit mTORC1 [84].

## 5. Prospects

Although, to date, there is no effective treatment for nervous system diseases, a wide range of natural small molecules with a variety of biological activities have shed bright light on a potential therapeutic strategy for these diseases. This review summarized a spectrum of neuroprotective potentials of fisetin involved in multiple aspects. In addition, the detailed modulatory potential of fisetin in different signaling pathways is discussed. Through the mediation of these multiple signal pathways by which fisetin exerts its neuroprotective role against neurological diseases, illustrated in Figure 3, fisetin achieves a neuroprotective effect. Moreover, fisetin has shown beneficial effects as a senolytic drug in preclinical models [144]. Encouragingly, fisetin promotes learning, memory and behavioral abilities through various molecular pathways. These characteristics make it a potential candidate for the future treatment of neurological diseases. Although fisetin has shown therapeutic potential for a variety of nervous system diseases, there are still many unresolved issues. For example, the low solubility and poor intestinal absorption of fisetin result in low bioavailability. Nevertheless, the preclinical data in cell and animal models have demonstrated that it is necessary to conduct more clinical trials to identify fisetin’s effects in humans. Therefore, in-depth pre-clinical trials are still needed to scientifically validate fisetin’s role in treating nervous diseases and translate potential health benefits into clinical applications.

## Data Availability

Not applicable.

## Abbreviations

AD	Alzheimer’s disease
PD	Parkinson’s disease
HD	Huntington’s disease
ALS	amyotrophic lateral sclerosis
TBI	traumatic brain injury
CNS	central nervous system
BBB	blood–brain barrier
Aβ	amyloid beta-peptide
NO	nitric oxide
iNOS	inducible nitric oxide synthase
TLR 4	Toll-like receptor 4
COX	cyclooxygenase
TNF-α	tumor necrosis factor α
GFAP	glial fibrillary acidic protein
PGD2	prostaglandin D2
PGE2	prostaglandin E2
HO-1	heme oxygenase-1
IL-1 β	interleukin 1 β
IL-6	Interleukin 6
MeHg	methyl mercury
LPS	lipopolysaccharide
ROS	radical oxygen species
NADPH	nicotinamide adenine dinucleotide phosphate
Nox	NADPH oxidase
BDNF	brain-derived neurotrophic factor
GDNF	Glial-cell-derived neurotrophic factor
MDA	malondialdehyde
GSH	glutathione
GCL	glutamate-cysteine ligase
GCLC	glutamate-cysteine ligase catalytic
GCLM	glutamate-cysteine ligase modifier subunit
SOD	superoxide dismutase
Nrf2	NF-E2 related factor 2
mTOR	mammalian or mechanistic target of rapamycin
MDA	malondialdehyde
Nrf2	NF-E2-related factor 2
Cdk5	Cyclin-dependent kinase 5
SNAP-25	synaptosomal-associated protein 25
PSD-95	postsynaptic density protein 95
CaMKII	Calcium–calmodulin (CaM)-dependent protein kinase II
NEP	neprilysin
CREB	cAMP response element-binding protein
AChE	acetylcholinesterase
NF-κB	nuclear factor kappa B
MAPK	mitogen-activated protein kinase
IKK	IκB kinases
ARE	antioxidant-response element
Keap1	Kelch-like ECH-associated protein 1
PI3K	Phosphoinositide 3-kinase
TFEB	Transcription factor EB
